# A single nucleotide substitution at the 3′-end of *SBPase* gene involved in Calvin cycle severely affects plant growth and grain yield in rice

**DOI:** 10.1186/s12870-020-02541-x

**Published:** 2020-07-22

**Authors:** Chun Li, Na Li, Rui Huang, Congping Chen, Jia Guo, Xiaorong Yang, Xiangyu Zhang, Changhui Sun, Xiaojian Deng, Pingrong Wang

**Affiliations:** State Key Laboratory of Crop Gene Exploration and Utilization in Southwest China, Rice Research Institute, Sichuan Agricultural University, 211 Huimin Road, Wenjiang District, Chengdu, 611130 China

**Keywords:** Rice (*Oryza sativa*), SBPase, Calvin cycle, Growth and development, Yield

## Abstract

**Background:**

Calvin cycle plays a crucial role in carbon fixation which provides the precursors of organic macromolecules for plant growth and development. Currently, no gene involved in Calvin cycle has been identified in monocotyledonous plants through mutant or/and map-based cloning approach.

**Results:**

Here, we isolated a low-tillering mutant, *c6635*, in rice (*Oryza sativa*). The mutant displayed light green leaves and intensely declined pigment contents and photosynthetic capacity at early growth stage. Moreover, its individual plant showed a much smaller size, and most individuals produced only two tillers. At mature stage, its productive panicles, grain number and seed setting rate were significantly decreased, which lead to a sharp reduction of the grain yield. We confirmed that a single nucleotide mutation in *LOC_Os04g16680* gene encoding sedoheptulose 1,7-bisphosphatase (SBPase) involved in Calvin cycle was responsible for the mutant phenotype of *c6635* through map-based cloning, MutMap analysis and complementation experiments*.* Sequence analysis suggested that the point mutation caused an amino acid change from Gly-364 to Asp at the C-terminal of SBPase. In addition, *OsSBPase* gene was mainly expressed in leaf, and the encoded protein was located in chloroplast. The mutation of *OsSBPase* could significantly affect expression levels of some key genes involved in Calvin cycle.

**Conclusions:**

We successfully identified a *SBP*ase gene in monocotyledonous plants. Meanwhile, we demonstrated that a single nucleotide substitution at the 3′-end of this gene severely affects plant growth and grain yield, implying that the Gly-364 at the C-terminal of SBPase could play an important role in SBPase function in rice.

## Background

The energy that human and other life on Earth depend on originally come from the Sun. Photosynthesis, as the sole biological process converting solar energy into chemical energy, is of great importance for human survival. Over 90 % of crop biomass is derived from photosynthetic products [1]. Photosynthesis efficiency of crop plants is a key factor for crop yield. Photosynthesis encompasses light reactions and dark reactions which also are called carbon fixation reactions. In the five carbon fixation pathways of autotrophs, Calvin cycle is the only biosynthetic process used in plants [2]. Photosynthetic carbon assimilation is inefficient in Calvin cycle of C3 plants, which is a limiting factor for crop yields [3, 4].

Calvin cycle is the first pathway in photosynthesis and assimilates atmospheric carbon dioxide into skeletons of organic compounds which are the precursors of organic macromolecules and the basics for plant growth and development [5, 6]. It occurs in the chloroplast stroma and comprises 13 enzymatic steps catalyzed by 11 enzymes proceeding in three distinct phases: carboxylation of ribulose 1,5-bisphosphate (RuBP), reduction of 3-phosphoglycerate and regeneration of RuBP [7, 8]. The first enzymatic reaction in Calvin cycle is carboxylation of RuBP. It converts CO_2_ and RuBP into two 3-phosphoglycerate and is catalyzed by ribulose bisphosphate carboxylase/oxygenase (Rubisco). Competitive inhibition of oxygen to Rubisco leads to low turnover rate of RuBP, so Rubisco is a key rate-limiting enzyme in photosynthesis [9–11]. Reduction of 3-phosphoglycerate into glyceraldehyde 3-phosphate consists of two enzymatic reactions catalyzed by 3-phosphoglycerate kinase (PGKinase) and glyceraldehyde-3-phosphate dehydrogenase (GAPDH). Regeneration of RuBP is a relative complex phase including 10 steps catalyzed by eight enzymes: triose phosphate isomerase (TPI), aldolase, fructose-1,6-bisphosphate aldolase (FBAase), transketolase, sedoheptulose 1,7-bisphosphatase (SBPase), ribulose 5-phosphate epimerase (RPE), ribose 5-phosphate isomerase (RPI), phosphoribulokinase (PRKase) [8]. In this phase, limited electron transport capacity for regenerative reactions is another reason for ineffective carbon assimilation in Calvin cycle [12]. FBAase and SBPase are two key enzymes in regeneration of RuBP. FBAase catalyzes two reactions: one is the conversion of glyceraldehyde 3-phosphate and dihydroxyacetone phosphate (DHAP) to fructose 1,6-bisphosphate, and the other is the conversion of erythrose 4-phosphate and DHAP to sedoheptulose 1,7-bisphosphate. SBPase catalyzes sedoheptulose 1,7-bisphosphate to sedoheptulose 7-phosphate and plays a vital role in assigning the assimilated carbon to the regenerative phase to regenerate RuBP or to starch biosynthesis out of the cycle [13, 14]. This particular role emphasizes the importance of SBPase in carbon fixation of Calvin cycle or photosynthesis [5, 15, 16].

The full-length cDNA or genomic DNA of *SBPase* gene has been isolated from various photosynthetic organisms. The first study cloning its full-length cDNA sequence was done in wheat (*Triticum aestivum*) through screening a wheat cDNA library using maize SBPase [17]. Thereafter, the full-length of *SBPase* gene from several organisms has been identified, such as *Arabidopsis thaliana* and *Chlamydomonas reinhardtii* which obtained cDNA or genomic DNA using library screening method [18, 19], and Mulberry (*Morus alba var. multicaulis*), rice (*Oryza sativa*) and cucumber (*Cucumis sativus*) which cloned cDNA sequence by RACE (rapid amplification of cDNA ends) [20–22]. In addition, *Arabidopsis* SBPase gene has been studied by T-DNA insertion mutant [7]. However, no study including rice and other monocotyledons has been reported to clone *SBPase* gene using mutant or/and map-based cloning approach.

In our study, a low-tillering mutant, *c6635*, was identified in rice. The mutant showed severely retarded growth and development and sharply reduced grain yield. Map-based cloning, high-throughput sequencing, MutMap analysis and complementation experiments indicated a single nucleotide mutation in *LOC_Os04g16680* gene encoding SBPase in Calvin cycle was responsible for the mutant phenotype*. OsSBPase* was mainly expressed in leaf and its encoded protein was located in chloroplast. In addition, expression levels of six key genes for Calvin cycle in *c6635* mutant were detected by qRT-PCR at seedling stage.

## Results

### The mutant shows sharply reduced grain yield due to severely retarded growth and development

**Fig. 1:**
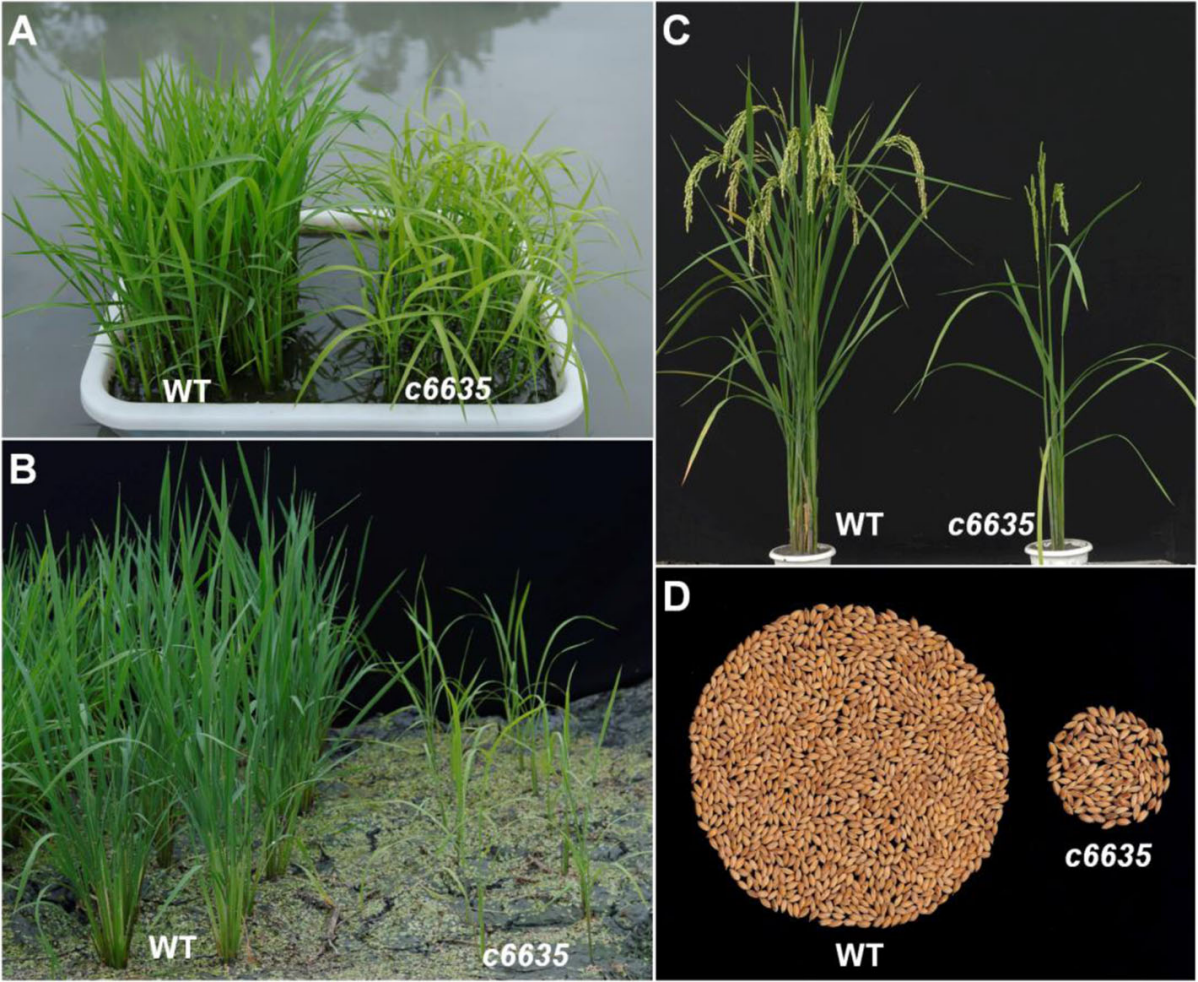
Phenotypic comparison between wild type and *c6635* mutant. The phenotype at seedling stage (**a**), tillering stage (**b**) and grain-filling stage (**c**). **d** The grain yield of each single plant

**Fig. 2:**
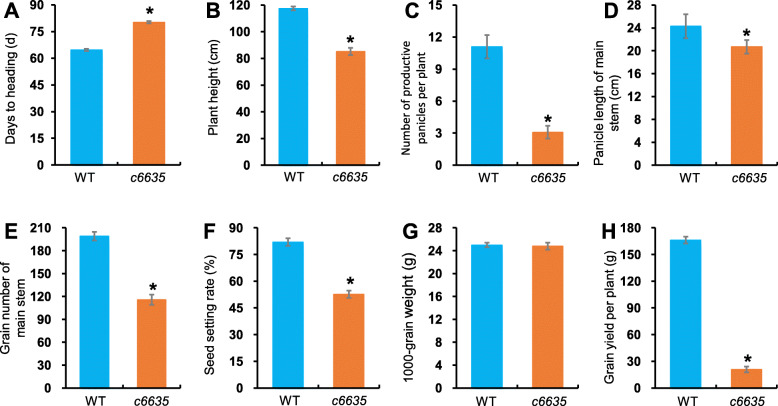
Comparison of major agronomic traits between wild type and *c6635* mutant. Error bars indicate SD and * *P* < 0.05

The leaves of *c6635* mutant displayed light green at early growth stage, and turned normal green as wild type at grain-filling stage (Fig. 1a, c). Moreover, its individual plant showed a much smaller size, and most individuals produced only two tillers (Fig. 1b, c). In addition, heading stage of the mutant was delayed by 15.6 days compared with wild type (Fig. 2a). As a result, most agronomic traits at mature stage were greatly affected in *c6635* mutant. For example, plant height, number of productive panicles per plant, grain number of main stem and seed setting rate significantly declined by 27.4, 67.5, 41.8 and 35.7%, respectively (Fig. 2b, c, e, f). Eventually, the grain yield of *c6635* was sharply reduced by 87.5% (Figs. 1d, 2h). The results suggested that the mutation in *c6635* severely affected plant growth and grain yield.

**Fig. 3:**
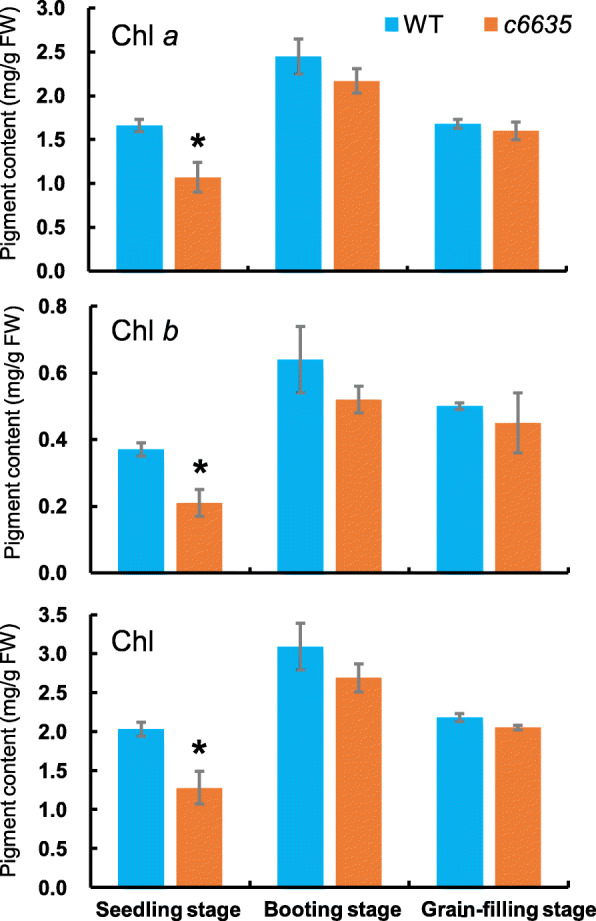
Comparison of pigment contents between wild type and *c6635* mutant at seedling, booting and grain-filling stages. Error bars indicate SD and * *P* < 0.05

To quantify the leaf colors of *c6635*, pigment contents of the mutant and wild type at seedling stage, booting stage and grain-filling stage were measured, respectively. Compared with wild type, the pigment contents including Chl *a*, Chl *b* and total Chl in the mutant were significantly decreased by 35.5, 43.2 and 36.5%, respectively, at seedling stage (Fig. 3). However, the pigment contents of the mutant at booting stage and grain-filling stage were still slightly reduced with no significant difference compared with wild type (Fig. 3). In fact, at grain-filling stage, the leaf color of *c6635* had turned normal green, and its pigment contents also had restored to the normal level of wild type. The results indicated that the light green leaf phenotype of *c6635* at seedling stage was due to the reduced pigment contents.

**Fig. 4:**
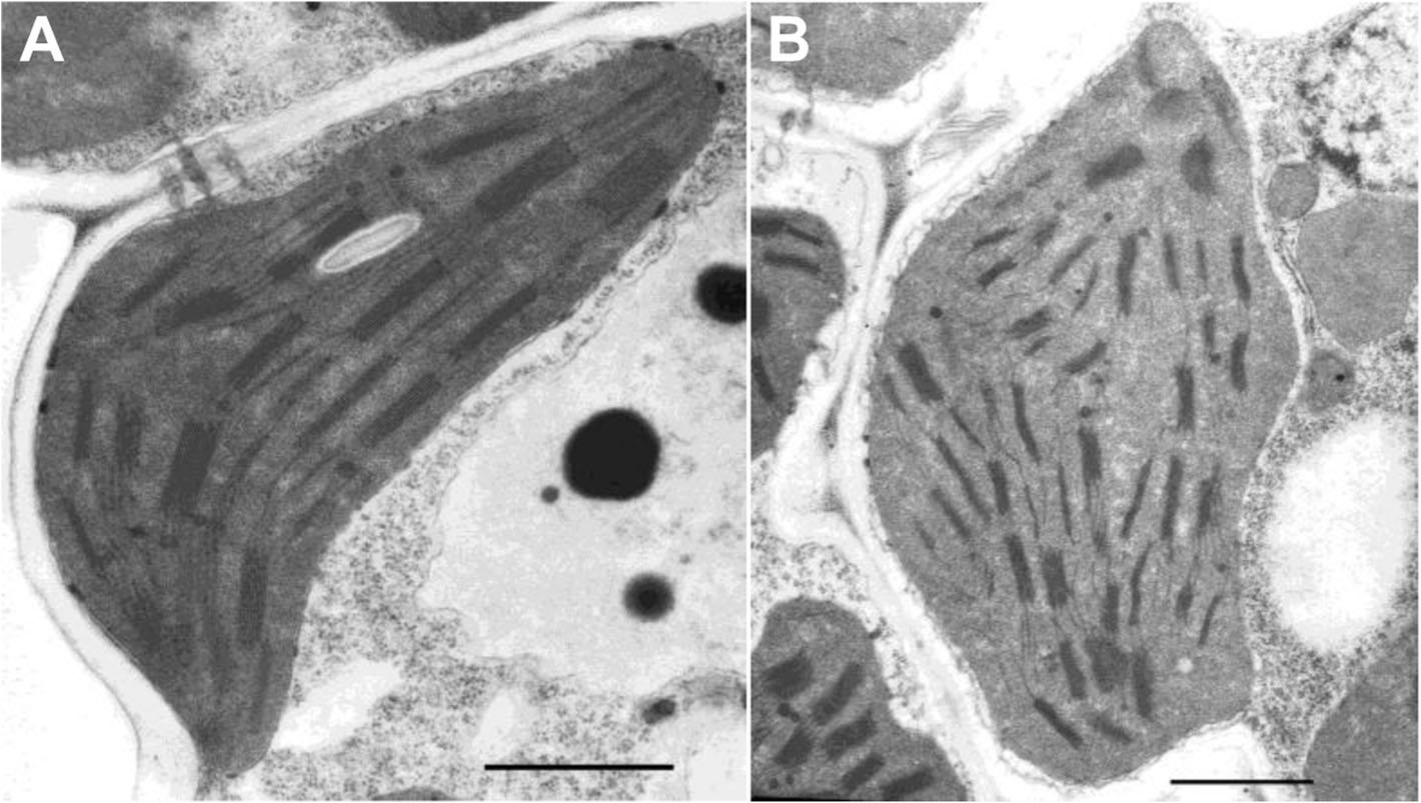
Chloroplast ultrastructure of wild type (**a**) and *c6635* mutant (**b**) at seedling stage. Scale bars equal l μm

In order to detect the development of chloroplasts in *c6635* mutant, its chloroplast ultrastructure at seedling stage were observed using transmission electron microscopy. The thylakoids of wild type were well stacked into grana (Fig. 4a), while the thylakoids of *c6635* were stacked with slight distortion (Fig. 4b). The results suggested that the development of chloroplast was somewhat suppressed in *c6635* mutant.

### Photosynthetic capability and starch contents of the mutant leaves intensely declined at early growth stage

**Fig. 5:**
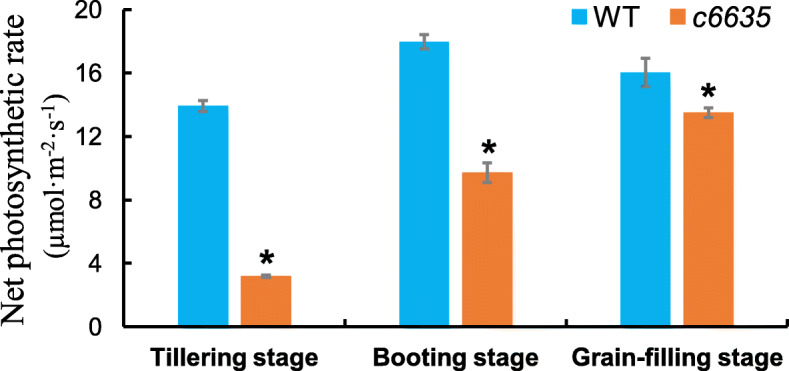
Net photosynthetic rate of wild type and *c6635* mutant at tillering, booting and grain-filling stages. The humidity in the chamber was about 30% at tillering stage, and about 50% at booting stage and grain-filling stage. Error bars indicate SD and * *P* < 0.05

In order to survey whether photosynthesis was affected in *c6635* mutant, net photosynthetic rate at various stages were measured with a portable photosynthetic apparatus. Net photosynthetic rates of *c6635* mutant at tillering, booting and grain-filling stages were significantly decreased by 77.1, 46.0 and 15.9%, respectively, compared with those of wild type (Fig. 5). The data of photosynthetic parameter indicated that the photosynthetic capability of *c6635* mutant was obviously deficient at early growth stage but gradually recovered at grain-filling stage.

**Fig. 6:**
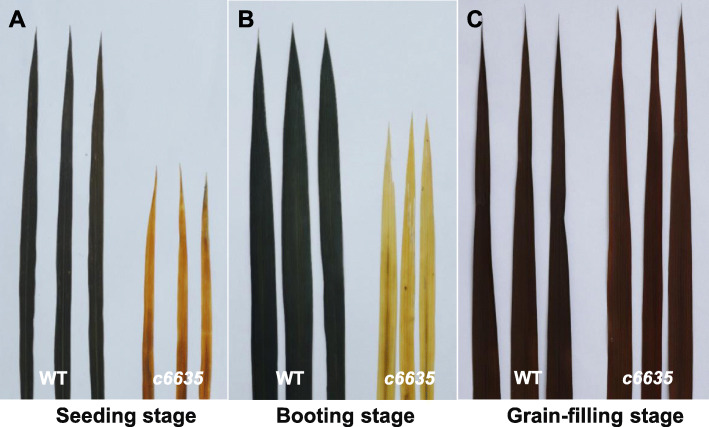
Potassium iodide-starch staining of leaves from wild type and *c6635* mutant at seeding stage (**a**), booting stage (**b**) and grain-filling stage (**c**)

To investigate whether starch accumulation was influenced in *c6635* mutant, we detected the starch contents in the leaves by starch iodine test. As shown in Fig. 6, dark brown or black were observed in the leaves of wild type at seedling and booting stages, whereas light brown or light yellow were observed in the leaves of *c6635* mutant at the same stages (Fig. 6a, b). At grain-filling stage, the leaves of both wild type and *c6635* mutant were stained into brown, although the leaves of wild type were still slightly deeper than that of *c6635* (Fig. 6c). The results indicated that only a little starch was accumulated in the leaves of *c6635* mutant at seedling and booting stages, but the starch accumulated in *c6635* leaves was almost recovered to the normal level of wild type at grain-filling stage, which was consistent with change of leaf color and net photosynthetic rate of the mutant.

### The *c6635* locus was mapped to a putative gene encoding SBPase involved in Calvin cycle

For genetic analysis of the mutant, *c6635* was crossed with its wild-type parent Zhonghua11 (*japonica* cultivar, hereinafter ZH11) to construct F_1_ and F_2_ populations. All plants of the F_1_ population showed normal leaf color and tiller number as wild type. Both normal and mutant phenotypes were observed in the F_2_ population and showed a good fit to the segregation ratio of 3:1 (χ^2^ < χ^2^_0.05_ = 3.84, *P* > 0.05). The results indicated that mutant phenotype of *c6635* was controlled by a single recessive gene.

**Fig. 7:**
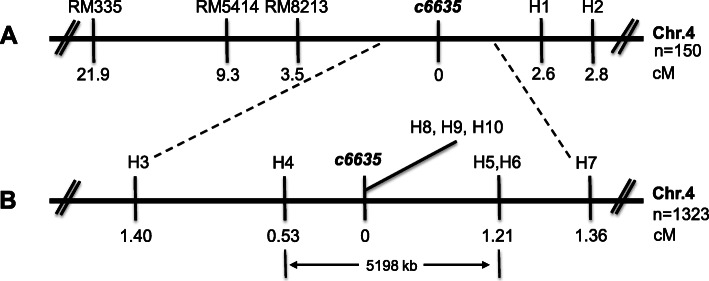
Preliminary mapping of the *c6635* locus. **a***c6635* was limited in a region between InDel marker H1 and SSR marker RM8213 on the short arm of chromosome 4. **b***c6635* was limited in a 5198-kb interval between InDel markers H4 and H5

Mapping population was derived from the cross between *c6635* and *indica* cultivar Gang46B (G46B). More than 300 SSR markers evenly distributing on the 12 chromosomes of rice were employed to carry out the preliminary mapping. Preliminary results showed that the mutant gene was located on chromosome 4 and was linked with RM335. Then SSR markers RM8213 and RM5414 and two newly designed InDel markers H1 and H2 (Table 1) were used for mapping, and *c6635* locus was located in a region between RM8213 and H1 on the short arm near the centromere of chromosome 4 (Fig. 7a). Subsequently, eight more InDel markers (Table 1) were used for further mapping. Finally, the *c6635* locus was limited into a region between H4 and H5 with a physical distance of 5200 kb (Fig. 7b).

**Table 1:** Polymorphic InDel markers developed for fine mapping in this study

Marker	Forward primer	Reverse primer
H1	TATGGGAAGGGACATTC	ATACTGTAGTTTCGAGGG
H2	CAACACATTGGTACTAGT	TAGGGATGGACAATAGGT
H3	TCCTTTGCCTTTCTCATT	AAGTCTAACCCTCCGATT
H4	CATGGACTTAGGTACTCT	GGTAGACAAACATTATTG
H5	ACACACCCTTAAGCTGCT	TTGCTACACATCTCCCAC
H6	CGCAACTTGATAACATAC	CTGTCTCAATCTAGGCAT
H7	TGGTATCGGAGATGAGTG	CCTGCCATCGCCGTGCAA
H8	GAATAAGAATCTACTCCCTC	CATCCCTAAATGAAACCTA
H9	TGTATGGCTGTCCTCTTG	TAAAGGAAGAAGAGCGAA
H10	TTGTCGCTGCTATGCTAT	TGAGAATGTGGGAACCAA

Due to the large physical distance between H4 and H5, high-throughput sequencing and MutMap method were used to identify the candidate gene in the region. High-throughput sequencing results of 25 mutant plants with light green leaf and fewer tillers from the (*c6635*/ZH11) F_2_ population showed that 14 SNPs between ZH11 and *c6635* genomes located within the 5200 kb-region (Additional file 1: Table S1). Among these SNPs, only two caused missense mutations: one was located in a gene (*LOC_Os04g18760*) encoding retrotransposon protein Ty3-gypsy subclass and the other was located in a gene (*LOC_Os04g16680*) encoding fructose-1,6-bisphosphatase, according to Rice Genome Annotation Project. The result from amino acid sequence alignment using BLASTP in NCBI showed that LOC_Os04g16680 was highly homologous to SBPase of *Arabidopsis thaliana*. Then, we amplified and sequenced the *LOC_Os04g16680* gene of the wild type and *c6635* mutant, and the result uncovered that a single nucleotide G substituted with A at position 2143 in genomic sequence leaded to an amino acid change from Gly-364 to Asp at C-terminal of the encoded protein. Therefore, we identified the *LOC_Os04g16680* gene as the candidate gene that was responsible for the mutant phenotype of *c6635*, and tentatively designated as *OsSBPase*.

**Fig. 8:**
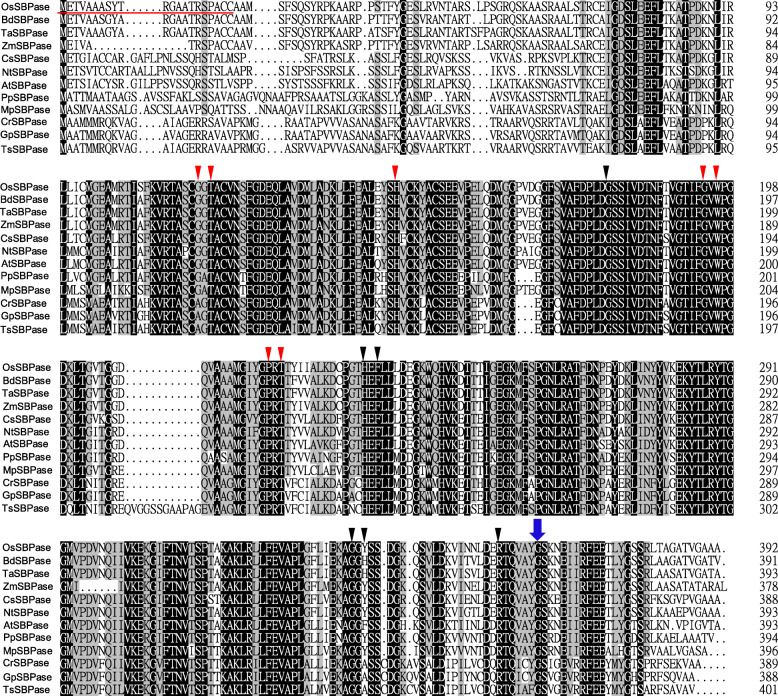
Homolog sequences of OsSBPase. Black shades indicate the identical residues and gray shades indicate the similar residues (≥75% identical). The blue arrow shows the mutation site of OsSBPase; A red underline shows the putative chloroplast signal peptides. The red triangles indicate the residues of the FBP/SBP domain and the black triangles indicate residues of the AMP domain. GenBank accession numbers for the respective protein sequences are as follows: OsSBPase (*Oryza sativa*, LOC_Os04g16680), BdSBPase (*Brachypodium distachyon*, XP_003564625.1), TaSBPase (*Triticum aestivum*, CBH32512.1), ZmSBPase (*Zea mays*, ONM36378.1), CsSBPase (*Cucumis sativus*, ACQ82818.1), NtSBPase (*Nicotiana tabacum*, AII99841.1), AtSBPase (*Arabidopsis thaliana*, AAB33001.1), PpSBPase (*Physcomitrella patens*, XP_024376141.1), MpSBPase (*Marchantia polymorpha*, ABF68592.1), CrSBPase (*Chlamydomonas reinhardtii*, XP_001691997.1), GpSBPase (*Gonium pectoral*, KXZ56517.1), TsSBPase (*Tetrabaena socialis*, PNH12434.1)

**Fig. 9:**
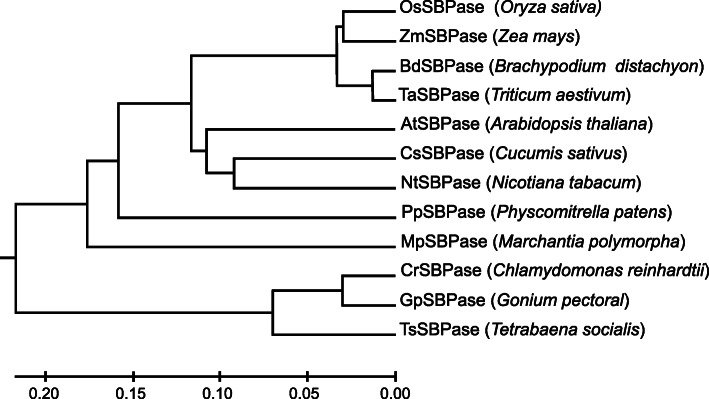
Phylogenetic analysis of OsSBPase and its homologs. GenBank accession numbers for the respective protein sequences are as Fig. 8

Sequence analysis revealed that *OsSBPase* gene contained eight exons and seven introns with 2750-bp genomic DNA and 1179-bp cDNA, and encoded a protein of 392 amino acids with a molecular mass of approximately 42.2 kD. A chloroplast transit peptide of 21 amino acid residues at N-terminus of OsSBPase protein was predicted by ChloroP and TargetP (Fig. 8). Multiple sequence alignment revealed that homologs of OsSBPase widely existed in monocotyledonous, dicotyledonous, moss and alga. Furthermore, OsSBPase had a high similarity to its homologs of monocotyledonous plants *Brachypodium distachyon*, *Triticum aestivum* and *Zea mays*, and dicotyledonous plants *Cucumis sativus*, *Nicotiana tabacum* and *Arabidopsis thaliana*, and moss *Physcomitrella patens* and *Marchantia polymorpha*, and alga *Chlamydomonas reinhardtii*, *Gonium pectorale* and *Tetrabaena socialis* with identity of 94.4, 93.7, 92.9, 83.2, 81.6, 74.4, 77.0, 75.2, 69.9, 69.3 and 65.7% (Fig. 8). Phylogenetic analysis indicated that OsSBPase had the closest relationship to homolog proteins from monocotyledonous plants (Fig. 9).

### Mutant phenotype of *c6635* was rescued by transformation with the wild-type *OsSBPase* gene

**Fig. 10:**
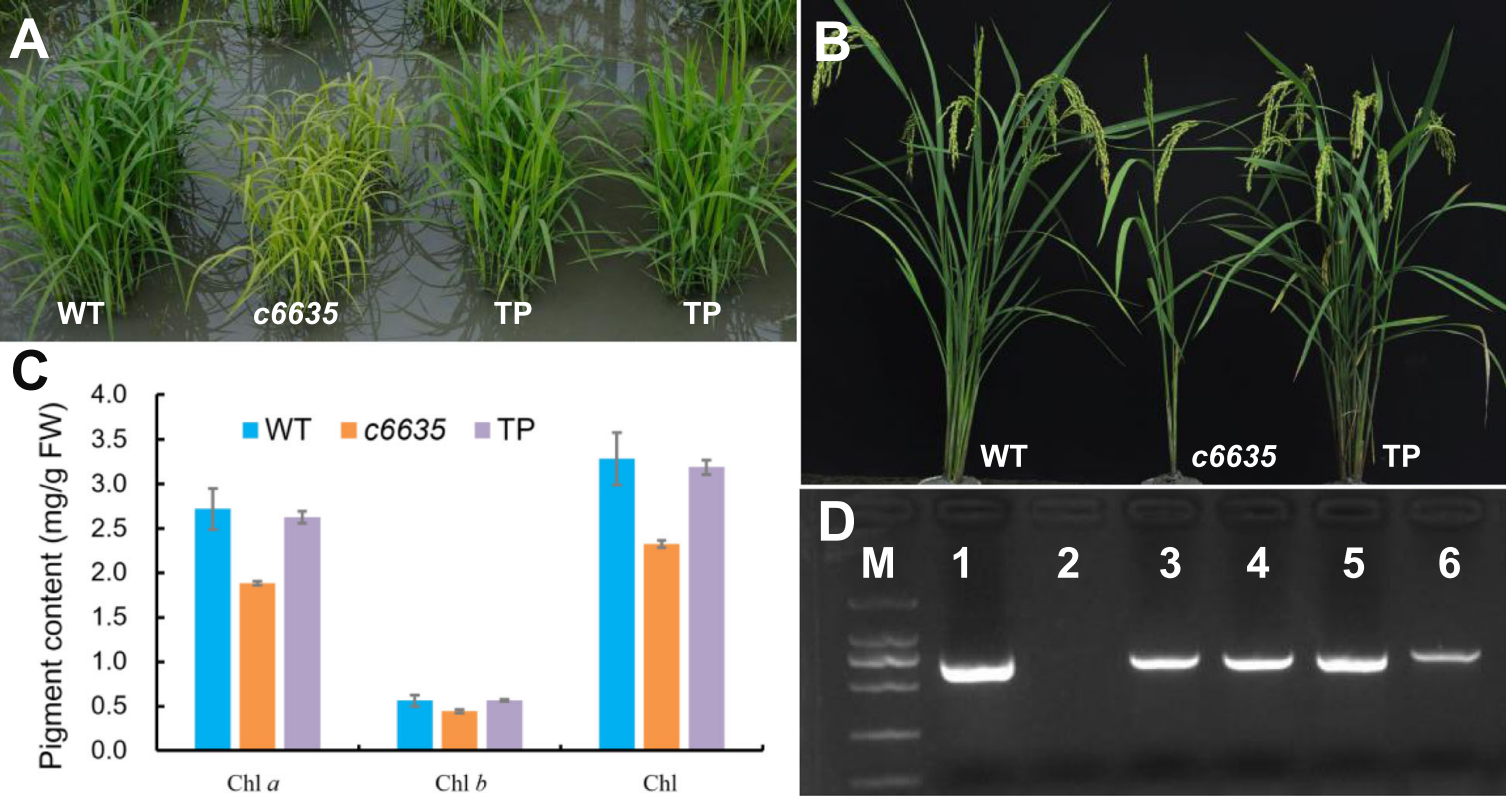
Complementation of *c6635* mutant with *OsSBPase* gene from its wild type. **a** Phenotypes of wild type (WT), *c6635* mutant, and PCR-positive transgenic plants (TP) at seedling stage. **b** Phenotypes of WT, *c6635* mutant, and TP plant at grain-filling stage. **c** Pigment contents of WT, *c6635* mutant and TP plants at seedling stage. Error bars indicate SD. **d** PCR test of PCR-positive transgenic lines (cropped from Additional file 2: Figure S1). M: DL-2000 marker; 1: PCR-positive control (pC2300-Actin-*OsSBPase* plasmid); 2: PCR-negative control (*c6635* mutant); 3–6: PCR-positive transgenic lines

In order to confirm our choice on candidate gene responsible for the mutant phenotype of *c6635*, genetic complementation experiments were carried out by transforming wild-type full-length cDNA of *OsSBPase* gene with *actin 1* promoter into *c6635* mutant. Finally, we got 19 independent transgenic lines, and PCR tests showed 17 lines were positive transgenic lines. As expected, normal leaf color and tiller number were observed in all the 17 positive transgenic lines (Fig. 10a, b, d). Moreover, pigment contents in transgenic lines showed no difference to wild type at seedling stage (Fig. 10c). The complementation results verified that the single nucleotide substitution in *OsSBPase* gene was responsible for the phenotype of *c6635*.

### OsSBPase protein was localized in the chloroplasts

**Fig. 11:**
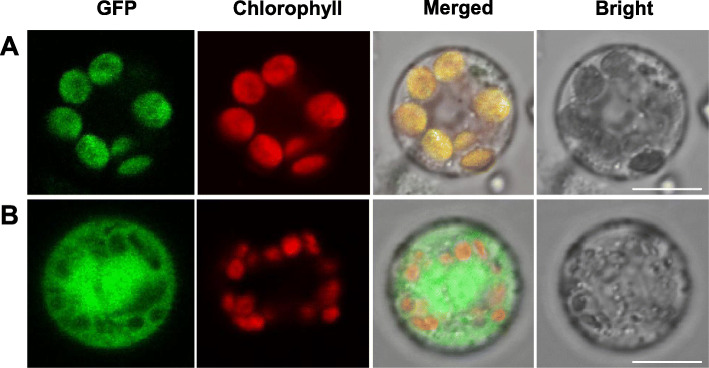
Subcellular localization of OsSBPase protein. **a** The OsSBPase-eGFP fusion protein. **b** The empty eGFP vector. Green fluorescence displays the exciting lights of GFP, red fluorescence displays the autofluorescence from chlorophylls, and yellow fluorescence displays the merged fluorescence from GFP and chlorophylls. Scale bars equal 10 μm

It was predicted that the OsSBPase protein contains a chloroplast transit peptide with 21 amino acid residues at its N-terminus using ChloroP and TargetP [23, 24]. To identify the actual subcellular localization of OsSBPase, the fusion protein OsSBPase-eGFP and green fluorescent protein (eGFP) were transformed and transiently expressed in rice protoplasts. The green fluorescence from OsSBPase-eGFP fusion protein exhibited the same pattern to red fluorescence from chlorophyll (Fig. 11a). However, green fluorescence from eGFP was observed throughout the protoplasts transformed with empty vector (Fig. 11b). The results confirmed that OsSBPase was located in chloroplast, being consistent with the place where it functions as a key enzyme for Calvin cycle. It should be noted that the mutation site in *c6635* mutant was out of the coding region of the chloroplast transit peptide and should not affect the localization of the mutated OsSBPase, although it affected the morphology of thylakoids, pigment contents, photosynthetic capability, biomass and grain yield.

### *OsSBPase* gene was mainly expressed in leaf

**Fig. 12:**
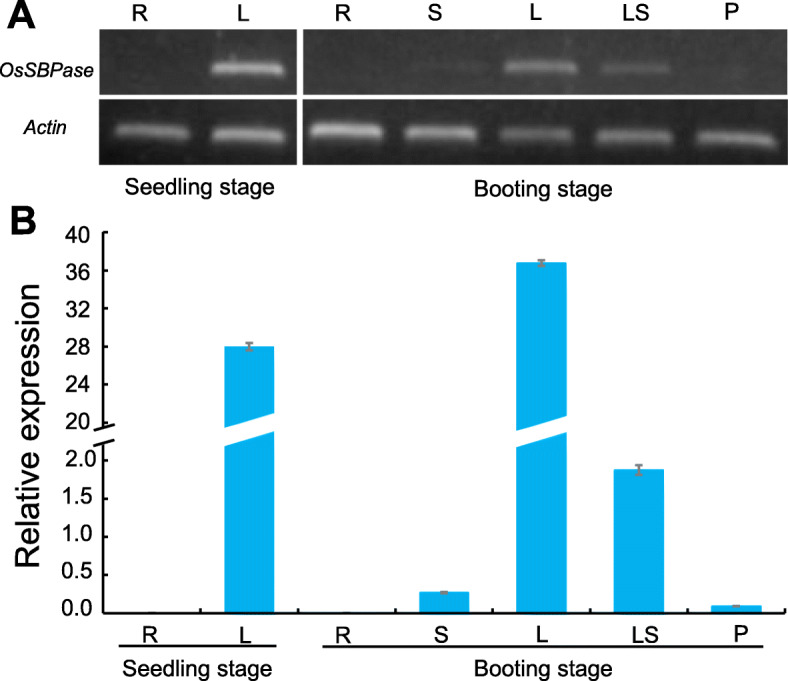
Expression pattern of the *OsSBPase* gene by semiquantitative analysis (**a**) (cropped from Additional file 3: Figure S2) and real-time quantitative analysis (**b**). R: Root, S: Stem, L: Leaf blade, LS: leaf sheath, P: young panicle. *Actin 1* was examined as the internal control. Error bars indicate SD

To investigate spatiotemporal expression pattern of *OsSBPase,* total RNA extracted from various tissues or organs of wild type at seedling and booting stages were used to conduct semiquantitative and quantitative RT-PCR analysis. Both tests showed the same spatiotemporal expression pattern that it had extremely high expressions in leaf blade at seedling and booting stages, high expressions in leaf sheath and slight expression in stem and young panicle at booting stage, but no detectable expression in root at both stages (Fig. 12). The results suggested that *OsSBPase* was mainly expressed in leaf and scarcely expressed in root.

### Expression levels of some key genes for Calvin cycle were affected in the mutant

**Fig. 13:**
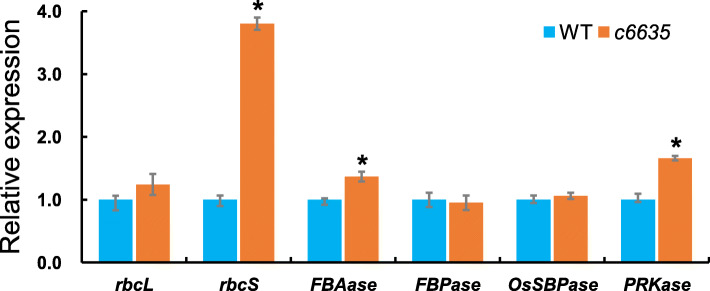
Expression analysis of six genes for Calvin cycle in wild type and *c6635* mutant at seedling stage. The expression level of each gene in the wild type was set to 1.0, and those in *c6635* mutant were calculated accordingly. Error bars indicate SD and * *P* < 0.05

To investigate the impact of *OsSBPase* mutation to the transcription of genes involved in Calvin cycle, we detected the expression levels of six key genes for Calvin cycle (*rbcL*, *rbcS*, *FBAase*, *FBPase*, *OsSBPase* and *PRKase*) by real-time quantitative PCR at seedling stage. *rbcL* and *rbcS* encode the large and small subunits of Rubisco which is a rate-limiting enzyme catalyzing the first reaction in Calvin cycle. FBAase catalyzes two reactions and determines the fate of dihydroxyacetone phosphate (DHAP) in Calvin cycle. FBPase catalyzes the conversion of fructose 1,6-bisphosphate to fructose 1,6-bisphosphate. PRKase catalyzes the conversion of ribulose 5-phosphate to ribulose 1,5-bisphosphate. As shown in Fig. 13, the six key genes showed different responses to the mutation of *OsSBPase* in *c6635* mutant. The expression levels of *rbcS*, *FBAase* and *PRKase* in *c6635* were significantly up-regulated compared to those in wild type, while the expression levels of *rbcL*, *FBPase* and *OsSBPase* showed no differentiation between *c6635* and wild type. Interestingly, *rbcS* showed the greatest expression difference with about fourfold between *c6635* and wild type. The results indicated that *OsSBPase* mutation had a significant effect on the expression of some key genes involved in Calvin cycle.

## Discussion

Up to now, *SBPase* genes of many species have been cloned using various methods and technologies [7, 18–22]. However, no *SBPase* genes of monocotyledonous plants have been cloned using mutants and/or map-based cloning methods. In the present study, we characterized a low-tillering mutant *c6635*. By map-based cloning and MutMap analysis, we found that *C6635* locus encoded sedoheptulose 1,7-bisphosphatase (OsSBPase) in rice. Further complementation analysis showed that the low-tillering phenotype of *c6635* mutant was rescued after introducing full-length genomic sequence of wild-type *OsSBPase* gene. Therefore, we have successfully identified a *SBPase* gene in monocotyledonous plants through mutant and map-based cloning approach.

Calvin cycle is the fundamental biological process which provides precursors of organic macromolecules and is the basics for plant growth and development [5, 6]. Consequently, reduced expression levels or mutations in Calvin cycle genes could cause retarded growth and development of plants [7, 25–31]. For example, antisense transgenic tobacco with 20% decrease in SBPase activity displayed reduced growth rates and chlorosis [27]. T-DNA insertion in chloroplastic *FBPase* gene caused fewer leaves, smaller size, lower growth rates, and decreased fresh weight in Arabidopsis [30]. Arabidopsis *sbp* mutant, which is deficient in *SBPase*, showed pale-green and more compact rosette leaves, and also much smaller plant [7]. In our study, *OsSBPase* mutant *c6635* showed light green leaves and intensely declined pigment contents and photosynthetic capacity at early growth stage. Moreover, its individual plant showed a much smaller size, and most individuals produced only two tillers. At mature stage, its productive panicles, grain number and seed setting rate were significantly decreased, which lead to a sharp reduction of the grain yield. Interestingly, *c6635* mutant recovered to normal levels in leaf color, pigment content and starch accumulation at grain-filling stage (Figs. 1c, 3, 6c), which was a differentiation between *c6635* and the two Arabidopsis T-DNA insertion mutants. The reason for the differentiation could be that *SBPase* and *FBPase* genes in Arabidopsis *sbp* and *cfbp1* mutants were nearly null loss-of-function alleles due to T-DNA insertion [7, 30], while a single nucleotide substitution at the 3′-end of *SBPase* in *c6635* mutant did not cause completely loss of function. Another explanation for the leaf-color recovery of *c6635* at grain-filling stage could be that FBPase with similar function may partly compensate for the deficiency of SBPase [7, 30].

In cyanobacteria, FBP/SBPase catalyses two separate enzymatic reactions dephosphorylating sedoheptulose 1,7-bisphosphate and fructose 1,6-bisphosphate, which are executed by two separate enzymes, FBPase and SBPase, in higher plants [32]. Based on the evolutionary relationship and high similarity among FBP/SBPase, FBPase and SBPase, the structure, domains and active sites of SBPase could be speculated from FBP/SBPase and FBPase [17]. Functional FBP/SBPase was a tetrameric structure including four monomers (C1, C2, C3 and C4) and two dimer pairs (C1/C4 and C2/C3) [33]. FBP/SBPases from *Thermosynechococcus elongatus* and *Synechocystis sp*. PCC 6803 had two regulatory domains: the FBP/SBP domain and the Adenosine monophosphate (AMP) domain, and all key residues of the two domains were identical between the two cyanobacteria [32, 33]. The AMP domain constituted with residues of two adjacent monomers and AMP helped to lock the four monomers into the stable enzyme [32, 34]. FBPase from *Porcus* kidney had two states: active state without AMP binding and inactive state with AMP binding. The two different active states differed by a rotation between the two adjacent monomers [34, 35]. In our study, an amino acid change from Gly-364 to Asp at the C-terminal of SBPase caused severely retarded growth and development, and sharply reduced grain-yield in the *c6635* mutant of rice (Figs. 1, 2, 6), suggesting that the Gly-364 could play an important role in SBPase function. The possible explanation for this speculation is that Gly-364 at the C-terminal of OsSBPase is located around a residue of the AMP domain, which was likely to affect the rotation between the two adjacent monomers and finally to remarkably reduce the activity of SBPase in *c6635* mutant.

qRT-PCR analysis showed that the expression levels of *rbcS*, *FBAase* and *PRKase* were remarkably up-regulated while *rbcL*, *FBPase* and *OsSBPase* expression had no obvious differences between wild type and *c6635* mutant. Interestingly, rbcL and rbcS were the two subunits of Rubisco. However, they showed different expression patterns in *c6635* mutant (Fig. 13). The different expression patterns of *rbcL* and *rbcS* may be result from their different functions in Rubisco. rbcL is the large subunit encoded by the chloroplast *rbcL* gene and rbcS is the small subunit encoded by the nuclear *rbcS* gene [11]. Catalytic activity of Rubisco is determined by rbcL subunit containing catalytic domain and regulated by rbcS subunit [36], which may result in the different expression patterns of *rbcL* and *rbcS*.

Transgenic plants overexpressing heterologous *SBPase* or *FBP*/*SBPase* genes showed increased levels of enzymes, enhanced photosynthesis and biomass yields [37–40], and few study have reported that transgenic plants overexpressing itself *SBPase* gene showed increased biomass yields [14]. In rice, overexpression of rice *SBPase* gene had no effect on biomass and yield, while enhanced the tolerance to high temperature and salt stress [41, 42]. We also overexpressed *OsSBPase* gene in *japonica* cultivar Nipponbare, and preliminary observation showed that overexpressing *OsSBPase* gene in rice did not significantly enhanced biomass and grain yield, so we did not go further to investigate the agronomic traits by measurement and statistics. According to the data reported, overexpression of *SBPase* might have a different reaction in different plant species, especially overexpressing itself *SBPase* gene might not provide an advantage to some plants [41, 42].

## Conclusions

We successfully identified a *SBP*ase gene in monocotyledonous plants. Meanwhile, we demonstrated that a single nucleotide substitution at the 3′-end of this gene severely affects plant growth and grain yield, implying that the Gly-364 at the C-terminal of SBPase could play an important role in SBPase function in rice.

## Methods

### Plant materials and growth conditions

The plant materials used in this study were originally from our lab. Rice low-tillering mutant *c6635* was derived from a mutant library (*japonica* cultivar Zhonghua11) mutagenized by ethyl methanesulfonate (EMS) and was genetically stable after several generations. The *c6635* mutant was crossed with *japonica* cultivar ZH11 and *indica* cultivar Gang46B (G46B) respectively, in order to construct F_1_ and F_2_ population for genetic analysis and gene mapping. During the natural rice planting season, all plant materials were grown in the paddy field in Wenjiang, Chengdu, China.

### Measurement of pigment contents

Fresh leaves at seedling, booting and grain-filling stages were collected to measure the pigment contents. 0.2 g leaves were cutted into pieces and extracted with 80% acetone for 48 h in the dark. UV-visible spectrophotometer (BioMate 3S, Thermo, USA) was used to measure the pigment contents at 663, 646 and 470 nm following the method from Lichtenthaler and Wellburn [43].

### Observation of chloroplast ultrastructure

Chloroplast ultrastructure of wild type and *c6635* mutant were observed by transmission electron microscopy (TEM). The fresh leaves at seedling stage from wild type and *c6635* mutant were collected in the paddy field and immediately fixed in 3% glutaraldehyde fixation solution. Then the samples were treated according to Li et al. [44] and finally observed and photographed with an H-600IV transmission electron microscopy (Hitachi, Japan).

### Measurement of photosynthetic capability

Net photosynthetic rate (P_n_) were measured with full-expanded top leaves or flag leaves of wild type and *c6635* mutant at tillering stage, booting stage and grain-filling stage with a portable photosynthetic apparatus (Li-6400, Li-COR Inc., USA) [45]. All measurements were conducted early in the morning from 9:00 to 9:30 a.m. in sunny days (when the solar radiation was still low). Each leaf from wild type and *c6635* mutant at the same stage was measured right after each other in a short period. All measurements were conducted under the environmental control settings: a photon flux density of 1200 μmol m^− 2^ s^− 1^, temperature of 30 °C and CO_2_ concentration of 400 ppm.

### Starch iodine test

Fresh leaves at seedling, tillering and grain-filling stages from wild type and *c6635* mutant were harvested, and destained in 95% ethanol for 12 h, then submerged in iodine/potassium iodide (2%/5%) solution overnight under the shading condition [7]. The samples were observed and photographed with camera (EOS 800D, Canon, Japan).

### Map-based cloning of the c6635 mutant gene

The *c6635* mutant was crossed with ZH11 and G46B, respectively, to construct F_2_ population for genetic analysis and mapping. One thousand three hundred twenty-three individuals with low-tillering phenotype were selected from the (*c6635* × G46B) F_2_ population for gene mapping. Three hundred fifty-one markers evenly distributing on 12 chromosomes from Gramene (http://www.gramene.org/microsat) were used for preliminary mapping. New insertion/deletions (InDel) primers were designed with Gramene and Primer5.0 based on the difference between ZH11 and G46B for further mapping. Because the region limited by gene mapping was too large to identify the candidate genes, high-throughput sequencing and MutMap method were used to identify the candidate gene. Leaves were collected from 25 low-tillering plants in the (*c6635* × ZH11) F_2_ population, cutted into small pieces and pooled in an equal ratio. Then the mixed samples were sent to Biomarker Company (Beijing, China) for high-throughput sequencing. Single nucleotide polymorphisms (SNPs) between genomes of ZH11 and *c6635* mutant were detected based on ZH11 genome as the reference genomic sequence.

### Sequence analysis

Candidate genes were identified from Rice Genome Annotation Project (http://rice.plantbiology.msu.edu/). *OsSBPase* gene was finally selected as the candidate gene based on the annotation of each putative genes and the phenotype of mutant. Homolog sequences of OsSBPase were downloaded from NCBI. DNAMAN version 6.0 was used to analyze the multiple sequence alignment and MEGA5 was used to conduct phylogenetic analysis using the UPGMA method [46].

### Complementation of the c6635 mutant

To confirm that the mutation in *OsSBPase* gene was responsible for the low-tillering phenotype of *c6635* mutant, complementation experiments were conducted with *OsSBPase* gene from wild type transferring into *c6635* mutant. The full-length cDNA of *OsSBPase* from wild type was amplified with specific primers (F1: AGGTCTAGAATGGAGACGGTGGCCGCG, R1: AGCCTGCAGTTAGGCGGCGGCGCCCAC) with an *Xba*I site at 5′-end and a *Pst*I site at 3′-end. The PCR products were digested with *Xba*I and *Pst*I and cloned into pC2300 binary vector under *actin 1* promoter. The pC2300-*OsSBPase* was transformed into *c6635* mutant by *Agrobacterium tumefaciens*-mediated transformation. All transformants were identified by amplification with specific primers (F2: TCAGTGTAGCATTCGACC, R2: CAGCAGCAACTTGGTCTC).

### Subcellular localization of OsSBPase

The chloroplast transit peptide and subcellular localization and were predicted by TargetP 1.1 server and ChloroP 1.1 server [23, 24]. To determine the actual subcellular localization of OsSBPase, transient expression of *OsSBPase* gene in rice protoplasts was carried out. The full-length cDNA of *OsSBPase* was amplified with specific primers (F3: AGGGGTACCATGGAGACGGTGGCCGCG, R3: AGCTCTAGAATAGGCGGCGGCGCCCAC) with a *Kpn*I site at the 5′-end and an *Xba*I site at the 3′-end. pC2300–35S-*OsSBPase*-eGFP vector was constructed with PCR product inserted into the pC2300–35S-eGFP vector, then transformed into rice protoplasts following the method of Zhang et al. [47]. Fluorescence signals in rice protoplasts were detected with a laser scanning confocal microscope (Nikon A1, Nikon, Japan).

### RT-PCR analysis

Different tissues and organs of wild type and *c6635* mutant were collected from 7:00–8:00 a.m. in the paddy field. Total RNA was extracted from tissues and organs with an RNA isolater kit (Vazyme). 2 μg of total RNA was used to obtain first-strand cDNA with HiScript II Q RT SuperMix (R223–01, Vazyme, China) according to its instructions. To detect the expression pattern of *OsSBPase* gene in different tissues and organs, semiquantitative and quantitative RT-PCR were performed using gene-specific primers with *actin 1* gene as the internal control. The expression levels of six key genes involved in Calvin cycle were detected at seedling stage. All qRT-PCR (10 μl) was conducted in the real-time PCR system (qTOWER^3^ G, analytikjena) with 5 μl ChamQ Universal SYBR Mix (Q711–02, Vazyme), 1 μl primer mix, and 1 μl cDNA. The *actin 1* gene was used as the reference gene. The assays were carried out with three technical and biological replicates respectively. All primers used in RT-PCR analysis were listed in Table 2.

**Table 2:** Primer sequences used for quantitative RT-PCR in this study

Gene	Forward primer	Reverse primer	Reference
*rbcL*	CTTGGCAGCATTCCGAGTAA	ACAACGGGCTCGATGTGATA	[48]
*rbcS*	CAGCAATGGCGGCAGGAT	AGGGCACCCACTTGGAACG	[48]
*FBAase*	CCTGAGCAAGTATCTGAC	GTTCATCGCGTTCAGGTTCTGC	
*FBPase*	CGATGAGCTTCATCGTCG	CAAGAACTCTCGACCTTC	
*OsSBPase*	GGAGAAGTACACATTGCG	AACAGGAGCCTCAGCTTG	
*PRKase*	CACATGTTCTTATCCTGGC	GGCTCTCAACATAGATAAGC	

## Supplementary information

**Additional file 1: Table S1.** The information of fourteen single nucleotide polymorphisms (SNPs) identified by MutMap in the target region.

**Additional file 2: Figure S1.** PCR test of PCR-positive transgenic lines. M: DL-2000 marker; 1 and 18: PCR-positive control (pC2300-Actin-*OsSBPase* plasmid); 2: PCR-negative control (*c6635* mutant); 3–6, 8–17, 19, 21, 22: PCR-positive transgenic lines; 7 and 20: PCR-negative transgenic lines.

**Additional file 3: Figure S2.** Expression pattern of the *OsSBPase* gene by semiquantitative analysis (two replications in a gel). R: Root, S: Stem, L: Leaf blade, LS: leaf sheath, P: young panicle.

## Data Availability

The datasets used and/or analysed during the current study are available from the corresponding author on reasonable request.

## Abbreviations

RuBP: Ribulose 1,5-bisphosphate
Rubisco: Ribulose bisphosphate carboxylase/oxygenase
rbcL: Large subunit of Rubisco
rbcS: Small subunit of Rubisco
FBAase: Fructose-1,6-bisphosphate aldolase
FBPase: Fructose 1,6-bisphosphatase
SBPase: Sedoheptulose 1,7-bisphosphatase
PRKase: Phosphoribulokinase
