# Supplementary material for: A single nucleotide substitution at the 3′-end of SBPase gene involved in Calvin cycle severely affects plant growth and grain yield in rice
Source: BMC Plant Biol. 2020 Jul 22;20:345. doi: 10.1186/s12870-020-02541-x (PMC7374905; doi:10.1186/s12870-020-02541-x)
Supplement: Supplementary file 1 — Additional file 1: Table S1. The information of fourteen single nucleotide polymorphisms (SNPs) identified by MutMap in the target region. [file 12870_2020_2541_MOESM1_ESM.doc]

**Additional file 1: Table S1** The information of fourteen single nucleotide polymorphisms (SNPs) identified by MutMap in the target region.

|  | Mutation | Mutation region | | Mutation type | | Locus name | | Gene annotation |
| --- | --- | --- | --- | --- | --- | --- | --- | --- |
| 1 | C→T | Downstream | - | | - | | | - |
| 2 | C→T | Downstream | - | | - | | | - |
| 3 | C→T | Intergenic region | - | | - | | - | |
| 4 | C→A | Downstream | - | | - | | - | |
| 5 | T→C | Coding region | Intron | | - | | - | |
| 6 | C→T | Coding region | Synonymous mutation | | - | | - | |
| 7 | C→T | Coding region | Intron | | - | | - | |
| 8 | G→A | Coding region | Missense mutation | | LOC_Os04g16680 | | Sedoheptulose-1,7-bisphosphatase, putative, expressed | |
| 9 | G→A | Downstream | - | | - | | - | |
| 10 | A→G | Coding region | Missense mutation | | LOC_Os04g18760 | | Retrotransposon protein, putative, Ty3-gypsy subclass, expressed | |
| 11 | G→A | Downstream | - | | - | | - | |
| 12 | A→T | Downstream | - | | - | | | - |
| 13 | A→G | Intergenic region | - | | - | | | - |
| 14 | T→G | Intergenic region | - | | - | | | - |
