# Supplementary figures and images for: A single nucleotide substitution at the 3′-end of SBPase gene involved in Calvin cycle severely affects plant growth and grain yield in rice
Source: BMC Plant Biol. 2020 Jul 22;20:345. doi: 10.1186/s12870-020-02541-x (PMC7374905; doi:10.1186/s12870-020-02541-x)

M 1 2 3 4 5 6 7 8 9 10 11 12 13 14 15 16 17

M 18 19 20 21 22

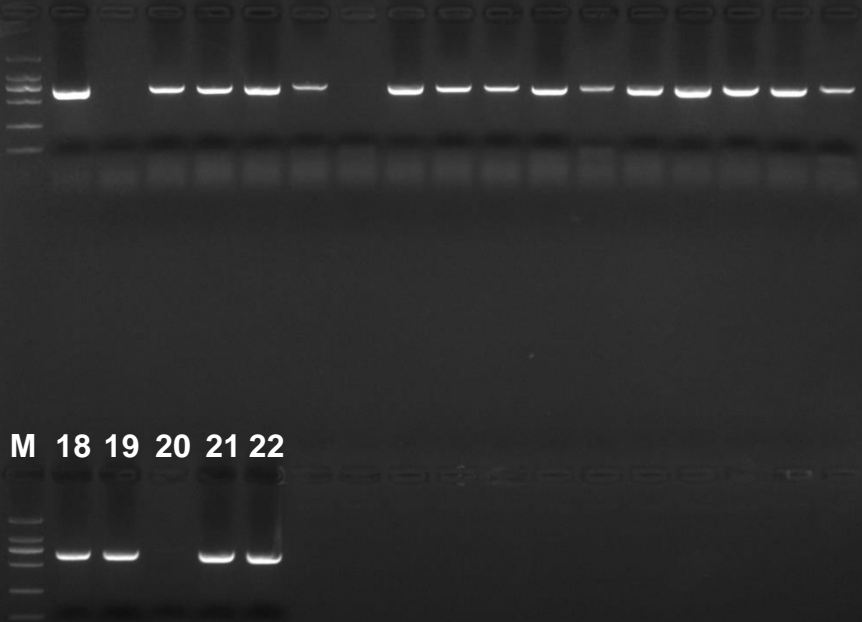

Supplement: Supplementary file 2 — Additional file 2: Figure S1. PCR test of PCR-positive transgenic lines. M: DL-2000 marker; 1 and 18: PCR-positive control (pC2300-Actin-OsSBPase plasmid); 2: PCR-negative control (c6635 mutant); 3–6, 8–17, 19, 21, 22: PCR-positive transgenic lines; 7 and 20: PCR-negative transgenic lines. [file 12870_2020_2541_MOESM2_ESM.pdf]

Two replications in a gel (left and right)

*OsSBPase*

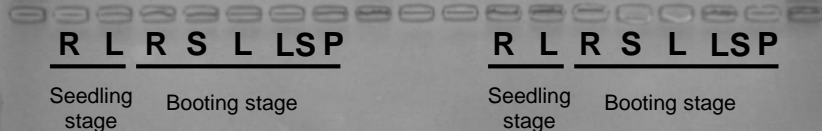

*Actin*

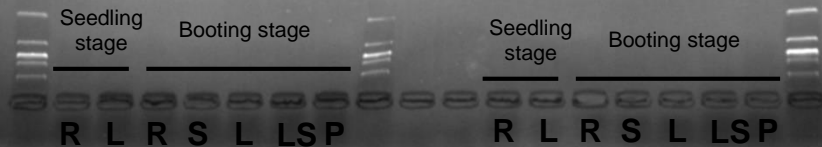

Supplement: Supplementary file 3 — Additional file 3: Figure S2. Expression pattern of the OsSBPase gene by semiquantitative analysis (two replications in a gel). R: Root, S: Stem, L: Leaf blade, LS: leaf sheath, P: young panicle. [file 12870_2020_2541_MOESM3_ESM.pdf]
